# Studying bacterial chemosensory array with CryoEM

**DOI:** 10.1042/BST20210080

**Published:** 2021-09-08

**Authors:** Zhuan Qin, Peijun Zhang

**Affiliations:** 1 Division of Structural Biology, Wellcome Trust Centre for Human Genetics, University of Oxford, Oxford OX3 7BN, U.K.; 2 Electron Bio-Imaging Centre, Diamond Light Source, Harwell Science and Innovation Campus, Didcot OX11 0DE, U.K.

**Keywords:** bacterial chemotaxis, chemoreceptor array, cryo-electron tomography, cryoEM, *in-situ* structure, subtomogram averaging

## Abstract

Bacteria direct their movement in respond to gradients of nutrients and other stimuli in the environment through the chemosensory system. The behavior is mediated by chemosensory arrays that are made up of thousands of proteins to form an organized array near the cell pole. In this review, we briefly introduce the architecture and function of the chemosensory array and its core signaling unit. We describe the *in vivo* and *in vitro* systems that have been used for structural studies of chemosensory array by cryoEM, including reconstituted lipid nanodiscs, 2D lipid monolayer arrays, lysed bacterial ghosts, bacterial minicells and native bacteria cells. Lastly, we review recent advances in structural analysis of chemosensory arrays using state-of-the-art cryoEM and cryoET methodologies, focusing on the latest developments and insights with a perspective on current challenges and future directions.

## Introduction

All motile bacteria and archaea examined to date possess a highly conserved chemosensory pathway that monitors the chemical environment and directs cell migration towards nutrient sources, a behavior known as chemotaxis [1–5]. For nearly six decades, bacterial chemotaxis has served as a paradigmatic model for the study of cellular sensory signal transduction and motile behavior and plays an important role in infection and disease in a variety of human, animal, and plant pathogens [6].

The signaling pathway for *E. coli* chemotaxis provides the best-characterized and most tractable system for elucidating the molecular mechanisms of intracellular signal transduction. Its chemotactic response features high sensitivity, wide dynamic range, extensive cooperativity and precise adaptation [3]. Those signaling functions are mediated by large chemosensory arrays that are typically localized across the cytoplasmic membrane at the cell pole. The periplasmic domains of the chemoreceptors detect attractant compounds and transmit stimulus information to their cytoplasmic domains to control the activity of the histidine kinase, CheA. Activated CheA donates phosphoryl groups to the messenger protein, CheY which governs the rotational sense of the flagellar motors. The chemoreceptors are also known as methyl-accepting chemotaxis proteins (MCPs) because they contain conserved glutamyl residues in their cytoplasmic signaling domain that are methylated and demethylated by enzymes CheR and CheB. Changes in MCP methylation state adjust the chemoreceptor's sensitive detection range.

**Figure 1.**
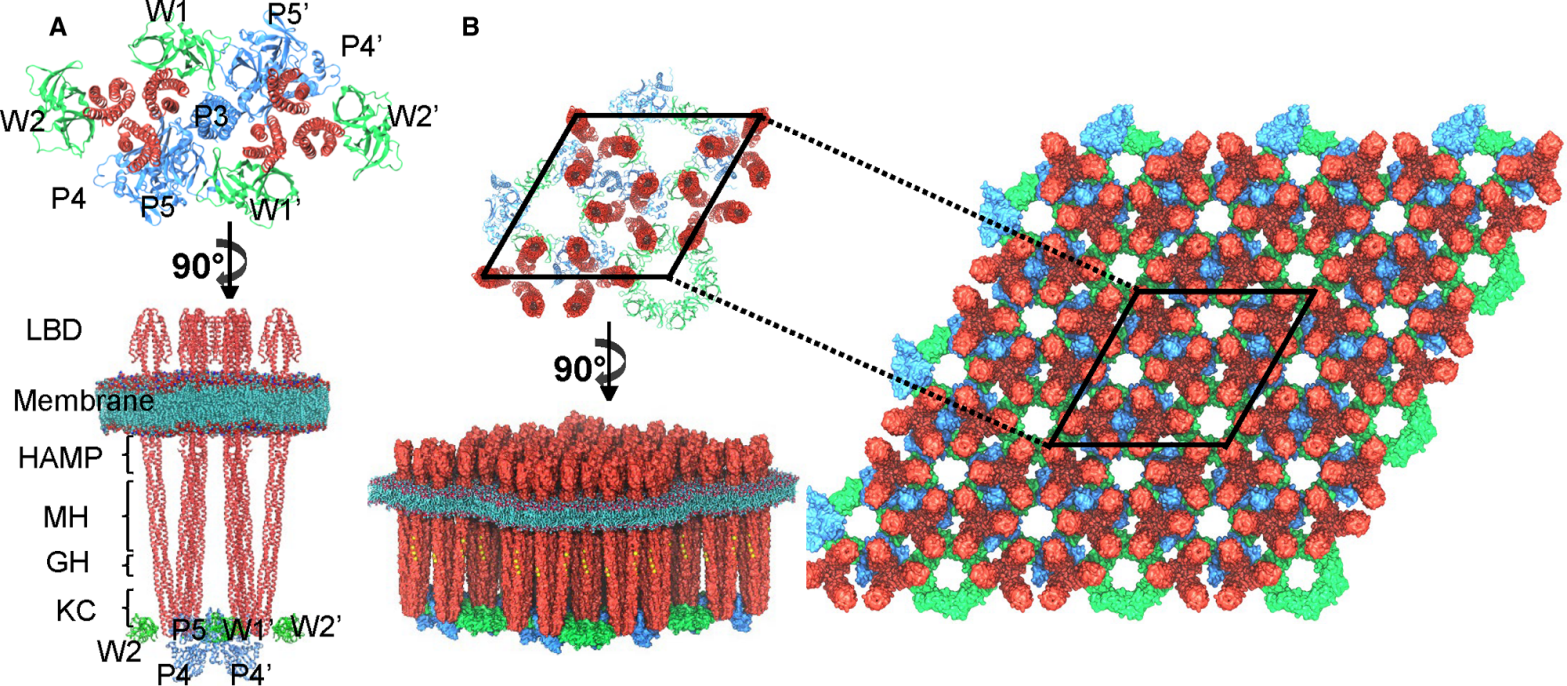
The chemoreceptor array model. (**A**) A core signaling unit (CSU) is composed of six receptor dimers, 1 CheA dimer and 2 CheW monomers (W1 and W1′). Two extra CheW (W2 and W2′) exist in the array forming the CheW ring, but they are not essential for the CSU activity. For the side view of the CSU (bottom left), from top to bottom there are ligand binding domain (LBD), membrane, HAMP (histidine kinase, adenylyl cyclases, methyl-binding proteins and phosphatases) domain, Methylation helix (MH) bundle, glycine hinge (GH), kinase Control (KC). (**B**) An extended array of 3 × 3 unit cells. The unit cell is enclosed by black lines. Chemoreceptor in red, CheA in blue and CheW in green.

The smallest assembly of chemotaxis proteins that is capable of key chemosensory functions, including kinase activation and control, is known as the core signaling unit (CSU) [7]. It is made up of two trimers of receptor dimers, a CheA dimer and two CheW monomers (Figure 1A). Two additional CheW molecules are present in the array forming the CheW rings (W2 and W2′ in the Figure 1A), but they are not essential for the CSU activity. There are five MCP-family receptors in *E. coli*, among which Tar (the aspartate and maltose receptor) and Tsr (the serine receptor) are the most abundant. The receptor proteins are homodimers of subunits ∼550 amino acids in length. The receptor molecule has three principal domains: a periplasmic ligand-binding domain (LBD), a four-helix bundle HAMP domain at the membrane-cytoplasm interface, and an extended four-helix coiled-coil containing the methylation sites and a hairpin tip that interacts with CheA and the coupling protein CheW (Figure 1A). CheA is a homodimer of ∼650-residue subunits comprising five functionally distinct domains (P1 to P5) [8]. P1 and P2 are connected to one another and the rest of the protein via flexible linkers [9]. P1 contains an autophosphorylation site, His48; P2 binds CheY and CheB to increase their local concentration near the phosphodonor P1 domain. P3 is a dimerization domain that has a fold similar to the receptor cytoplasmic domain dimer [10]. P4 contains essential catalytic residues, including an ATP binding site. The P5 domain is structurally homologous to CheW [11]. P5-W interactions in the CSU serve to couple CheA activity to receptor control.

The bacterial chemotaxis machinery has been extensively studied using a battery of genetic, biochemical, biophysical, and structural methods [12–14]. In recent years, high-resolution cryo-electron microscopy (cryoEM), especially cryo-electron tomography (cryoET) have provided potent approaches to study the organization of the chemosensory array both *in vitro* and *in vivo*. The structure and function of chemoreceptors and signaling arrays have been studied in a variety of systems (Table 1), including nanodiscs [7,15,16], lipid monolayer arrays [17,18], bacterial minicells [19,20], lysed bacterial ghosts [21,22] and intact native bacterial cells [1,20,23–25]. Great strides have been made in understanding the architecture of the chemosensory array and the detailed structure of the CSU. In this review, we present an overview of systems employed for structural analysis of chemosensory arrays by cryoEM and cryoET and review advances made during the past few years, highlighting some exciting cases where sub-nanometer resolution has been achieved and novel functional insights have been obtained.

**Table 1:** List of bacterial systems in which MCPs were studied by cryoET

System	Structures released	References
*E. coli* (UU1607)		[38]
*C. crescentus* (CB15N)		[39]
*C. crescentus* (CB15N)		[47]
*E. coli* (HCB721)		[40]
*C. crescentus* (CB15N), *E. coli* (RP437 and MG1655), *T. maritima* (MSB8/DSM 3109), *V. cholerae* (TRH7000), *M. magneticum sp.* (AMB-1), *H. hepaticus* (ATCC 51449), *C. jejuni* (ATCC 29428), *R. sphaeroides* (NCIB 8253), *B. burgdorferi* B31 cells (ATCC 35210), *L. monocytogenes* (strain 10403S)		[1]
*S. enterica* minicells (TH17261), lysed *E. coli* and B. subtilis cells, and intact *H. hepaticus* cells	EMD-2158	[20]
*E. coli* minicells (WM4011)	EMD-5404	[19]
*E. coli* (RP437)	EMD-2414, EMD-5545, EMD-5546, EMD-5547, EMD-5548, EMD-5549, EMD-5550, EMD-5716	[32]
Lysed *R. sphaeroides* (JPA543OE, etc.)		[48]
*E. coli* (RP437, UU2619, UU2564, HCB326, CO4)		[12]
*In vitro* reconstitution of chemotaxis 2D array	EMD-3234, EMD-6319, EMD-6320, PDB-3JA6	[17]
*V. cholera* (PM7, pPM045, PM15, PM16, PM049, PM22)	EMD-3398	[49]
*H. pylori* (7.13)	EMD-8460	[23]
*In vitro* construction of Tar in nanodisc		[15]
*S. enterica* minicell (TH16943)		[50]
Lysed *V. cholerae* (C6706)		[22]
*A. brasilense*		[24]
Lysed *E. coli* (RP437)	EMD-4991, EMD-4992, EMD-4993	[33]
In vitro reconstitution of chemotaxis 2D array	EMD-10050, PDB- 6S1K	[18]
*E. coli* minicells (WM4196)	EMD-10160	[28]
*T. denticola* (Td) ATCC 35405	EMD-11381, EMD-11384, EMD-11385, EMD-11386	[25]
*V. cholerae* (C6706, PM6, PM7), *P. aeruginosa* (PAO1), *S. oneidensis* (MR-1), and *M. alcaliphilum* (20Z^R^)		[51]
*E. coli* minicell (WM4196)		[37]

## Nanodisc-embedded chemoreceptors

**Figure 2.**
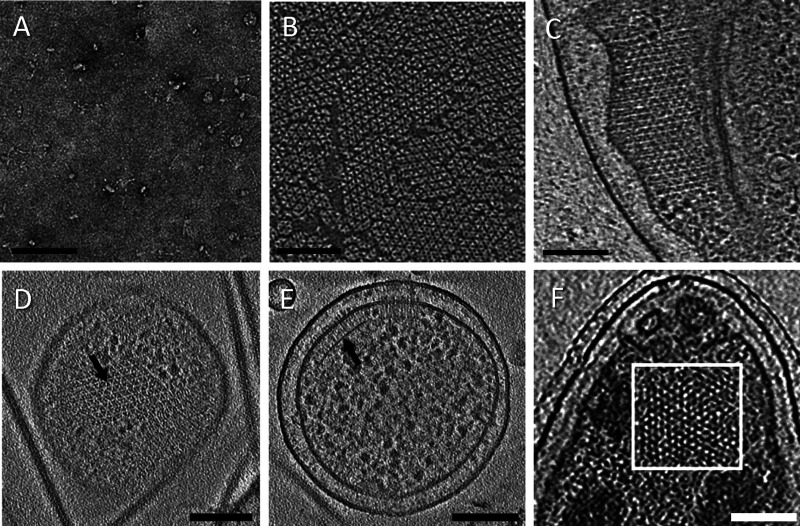
Overview of systems for structural analysis of chemoreceptor arrays by cryoEM and cryoET. (**A**) Nanodisc embedded Tar homodimer. (**B**) *In vitro* reconstituted chemoreceptor arrays on 2D lipid monolayer. (**C**) Phage φX174 E gene lysed ghost *E. coli* cell. (**D** and **E**) *E. coli* minicell. (**F**) Polar region of an intact Caulobacter swarmer cell. Scale bars, 100 nm. Panels are reproduced from [15,17,21,28,47] with permissions.

Nanodiscs are commonly used for structural studies of membrane proteins in the lipid bilayer environment. Chemoreceptor Tar dimers and trimers of dimers have been successfully incorporated into nanodiscs (Figure 2A) [15,16,26]. One receptor molecule in a nanodisc could bind ligand and transmit conformational changes to the cytoplasmic methylation sites, yet single receptor molecules cannot activate and control the kinase CheA [16]. Biochemical measurements suggest that two nanodiscs each containing a trimer of receptor dimers are needed to assemble a functional CSU, which has a stoichiometry of six receptor dimers, a CheA homodimer and two CheW monomers [7].

**Figure 3.**
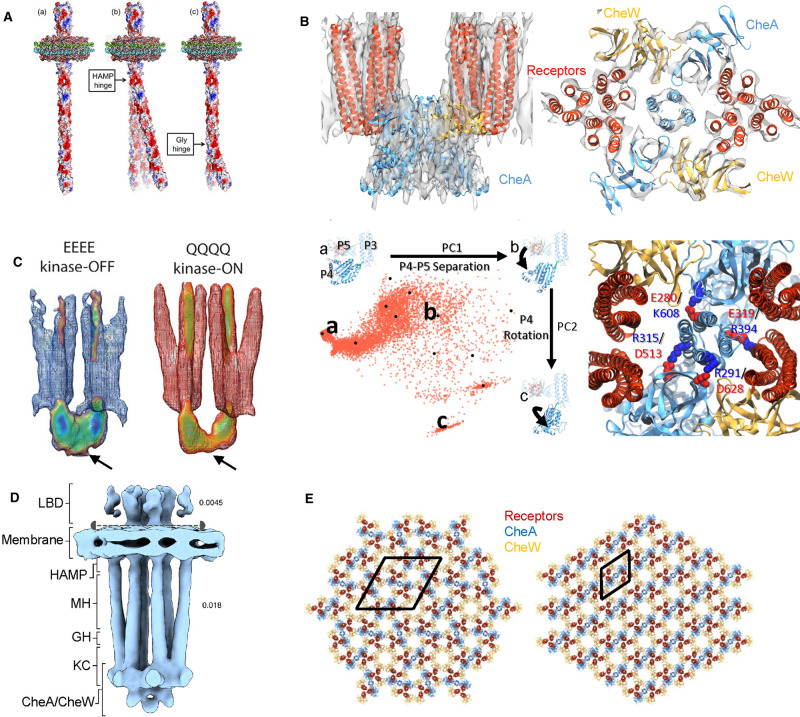
Structural analyses of chemoreceptors and arrays. (**A**) The shape of nanodisc embedded Tar homodimer was characterized by single particle analysis and ET. Two flexible hinges was observed: HAMP hinge and the glycine hinge. (**B**) (Top) CryoET STA map of the CSU from *in vitro* reconstituted monolayer arrays at 8.4 Å resolution in top view and side view, overlaid with an MDFF-derived atomic model. Receptor in red, CheA in blue and CheW in gold. (Lower left) The ensemble of P4 conformations (red dots) using generalized simulated annealing (GSA). There are 12 medoid structures (black dots) from clustering of the ensemble, three of those are labeled as a, b and c, shown in the figure, illustrating the rigid body rotation of P4–P5 separation and P4 rotation. (Lower right) CheA-P3 interactions CheA-P5 (R315/D513, E280/ K608, R291/D628) and Tsr receptor (E319/R394). Basic amino acids are in blue, and acid amino acids are in red. (**C**) CryoET STA of CSU from lysed *E. coli* cells containing Tsr_EEEE and Tsr_QQQQ arrays, suggesting a ‘keel’ density (arrow) might be CheA-P1-P2 domains, and it is more mobile in kinase-on states (QQQQ). (**D**) CryoET STA of CSU from *E. coli* minicells containing wild-type arrays. Receptor domains are labeled as in Figure 1. The map was shown at different thresholds (0.0045 top and 0.018 bottom) separating by the dashed line. (**E**) Alternative chemosensory array lattice arrangement found in *E. coli* minicell, shown with atomic models (colors indicated in the key). Both the original and the alternative lattices are P2 lattices, with a unit cell containing three CSUs (original, left) or a single CSU (alternative, right). Panels are reproduced from [15,18,33,28,37] with permissions.

The shape of a nanodisc-embedded Tar chemoreceptor was characterized by negative stain single particle analysis and electron tomography [15]. The molecule exhibited two flexible hinges (Figure 3A), one located at the membrane-distal boundary of the HAMP domain, another near conserved glycine residues midway between the methylation sites and the hairpin tip. The EM results suggested that the dimer could bend at the both hinge from 0° to ∼20°, and that such bending may facilitate chemoreceptor packing in the higher-ordered array.

## 2D lipid monolayer array

A chemosensory array reconstituted on a lipid monolayer provided high-resolution structural information (Figure 2B) [17]. Three soluble Tar signaling cytoplasmic fragments (TarCF) with different combinations of Q and E residues at the four methylation sites were studied in this manner: QEQE, the wild-type pattern; QQQQ which has a kinase-ON conformation; and EEEE, which has a kinase-OFF conformation [17]. Using cryoET and subtomogram averaging (STA), where repeating subvolumes, such as the CSUs within an array, are aligned and averaged, a 3D density map of the TarCF-QEQE CSU was reconstructed at 11.3 Å resolution [17], a significant improvement from previous attempts [19–21]. The CheA-P3 domain was readily visible in the density map. This study showed that the array contained both three-fold symmetric CheA–CheW rings and six-fold symmetric CheW only rings at the baseplate. Molecular dynamics (MD) simulations that optimized fitting of the atomic models to the density map revealed several conformations of the CheA-P4 domain that might be alternative activity states. By developing a new cryoET STA software, emClarity [27], the structure of the CSU was further improved to sub-nanometer resolution, revealing alpha helices for the first time (Figure 3B, top) [28]. This structure allows more precise MD modeling and comprehensive analysis of the CheA conformational landscape (Figure 3B, lower left) and detailed interactions between CheA-P3 and CheA-P5 and between CheA-P3 and the receptor (Figure 3B, lower right) [28]. Furthermore, comparison of cryoET STA structures of arrays composed of TarCF with QEQE, QQQQ and EEEE sequences suggest the stability of the array varies from highest with QQQQ to lowest with EEEE [18], consistent with other biophysical, biochemical and functional measurements [29,30].

## Lysed bacterial ghosts

An important limitation for structural analysis by cryoEM and cryoET is the requirement of a thin sample, typically less than 300 nm. Native *E. coli* cells are usually too thick to extract detailed structural information. One strategy to reduce the sample thickness while keeping the membranes and chemosensory arrays intact is creating flattened bacterial ghost cells where most of the cytoplasm is released. Bacterial ghost cells are usually made by treating cells with antibiotics such as penicillins or lysozyme-EDTA [31].

Briegel and colleagues took this approach to study the chemosensory array in *E. coli* cells and *T. maritima* cells [1,32]. The cryoEM images of lysed cells showed that the basic arrangement of the transmembrane chemosensory array is a ‘trimer-of-dimer' receptor organization that is universal across bacterial species surveyed [1]; Moreover, the array remains ordered in different signaling states [32]. The CSU maps of receptors locked in the kinase-ON and kinase-OFF states revealed a ‘keel' density that likely represents the P1-P2 domains of CheA, which seem to be more mobile in kinase active states (Figure 3C) [13,32]. Study of CSU from Tsr_EEEE (kinase-OFF) and Tsr_QQQQ (kinase-ON) arrays from lysed cells using Molecular Dynamics Flexible Fitting (MDFF) of the atomic model into the density map, showed that the trimer of dimer changes from a compact conformation in Tsr_EEEE to an expanded conformation in the Tsr_QQQQ [33]. However, the resolution of these averaged maps was limited to 20 ∼ 30 Å, preventing the visualization and distinction of CheA domains P3 and P5, and CheW. Neither was the cell membrane bilayer visible in these averaged maps. Thus, higher resolution structures of defined signaling states are needed to understand the mechanism of CheA regulation.

Another approach to make bacterial ghost cells is by exploiting the small phage lysis proteins that bacterial phages use to break out in a timely manner [34,35]. Both penicillin and small phage lysis proteins induce cell lysis by inhibiting cell wall synthesis, albeit at different stages. Using an inducible phage φX174 lysis protein E, localized cell lysis (or spot-lesion) can be triggered minutes before plunge-freezing for cryoEM sample preparation [21]. The lysis events were frequently captured during cryoEM sample vitrification, when ghost cells were seen sitting in a pool of ribosomes just being released from the cells. The bacterial membranes containing chemosensory arrays are largely intact in a hydrated environment (Figure 2C) [21].

## The minicell system

An alternative strategy to reduce sample thickness is to use cell strains that bud off small, genome-free minicells containing chemosensory arrays (Figure 2D,E). Most minicell-producing mutants are defective in a multiprotein system known as the Min system, resulting in asymmetric cell division [36]. Small daughter cells budded from the cell pole can be isolated and concentrated by differential centrifugation before plunge-freezing.

As minicells can be produced from a variety of bacteria through similar genetic modification, this technique provides a valuable option for structural study of membrane proteins *in situ* using cryoET. The average diameter of *E. coli* minicells is roughly 400 nm [28], which necessitates time-consuming searches to find subjects suitable for cryoET data collection. Recently, however, a cryoET STA structure of the full length CSU from an *E. coli* minicell was reported at 16 Å (Figure 3D) [28]. The density map shows the ligand binding domain, the membrane bilayer, the cytoplasmic domain of chemoreceptors and the baseplate where CheA and CheW are localized. The ligand biding domains has a much weaker density compared with the rest of the CSU and thus requires lowering the threshold to display (Figure 3D). The secondary structure features such as α-helices and β-sheets were not yet resolved.

The architecture of the chemosensory array was deduced by fitting atomic structures of the various component proteins into the EM map. The three-fold symmetric CheA/CheW rings and six-fold symmetric CheW only rings are located alternatively between tips of the receptor trimers (Figure 3E, left) [19,20]. The array is a P2 packing of CSUs containing three CSUs in the unit cell (Figure 3E, left). This assembling was also reported in *in vitro* construction of 2D monolayer arrays and lysed *E. coli* cells [17]. Recently, an alternative P2 packing with a unit cell containing a single CSU has been reported from native *Treponema denticola* [25] and an *E. coli* minicell [37] (see Figure 3E, right). Although the average map of CSU from the alternative packing was not reported, its basic structure appears to be conserved.

## Native bacteria cells

Chemosensory arrays have been directly visualized by cryoET near the cell edge or pole of intact *E. coli* cells [38]. Chemosensory arrays were also identified in intact *C. crescentus* cells (Figure 2F) [39]. The architecture of the array reported in these early studies was not clear [39,40], mainly due to the low signal and low contrast in the cryoET data because of the thickness of the intact bacterial cell.

Some bacterial cells are naturally thin and suitable for cryoET analysis on their own. Spirochetes, such as *Leptospira, Treponema,* and *Borrelia*, have a slim and elongated body shape with 200–300 nm diameter, making them good candidates for structural studies of chemosensory arrays by cryoET [25,41]. However, the chemotaxis machinery in spirochetes is more complicated and less-well studied than that of *E. coli*. For example, *T. pallidum* has one copy of most chemotaxis genes (*cheA*, *cheX*, *cheD*, *cheY*, *cheB* and *cheR*), but it has two copies of *cheW* [42]. *B. burgdorferi* has several copies of chemotaxis genes (2 *cheA*, 3 *cheW*, 3 *cheY*, 2 *cheB* and 2 *cheR*) [42]. Many questions regarding regulation and expression of the chemotaxis genes in these organisms have not been answered, making them less favorable subjects for structural study.

## Competing Interests

The authors declare that there are no competing interests associated with the manuscript.

**Table 2:** List of recently released cryoET software packages

Software	Description	References
IMOD	Tilted series alignment, CTF, 3D-CTF for cryo-ET	[52]
NovaCTF	3D-CTF for cryo-ET	[53]
Dynamo	Subtomo averaging and classification	[54]
emClarity	CTF, 3D-CTF, Subtomo averaging and classification	[27]
EMAN2.3	Tilted series alignment, CTF, 3D-CTF, Subtomo averaging and classification	[55,56]
Warp-RELION-M	Tilted series alignment, CTF, 3D-CTF, Subtomo averaging and classification	[57]

## Perspectives

- The bacterial chemosensory array has attracted a great deal of scientific interest as it plays an essential role in bacterial motility, is a virulence factor in pathogenic microbes, and serves as a paradigm for cellular sensory signal transduction and motile behavior. A structural knowledge of the array organization and precise interactions between the signaling components is essential to understanding the underlying molecular mechanisms of chemosensory array assembly, activation and high cooperativity, it is essential to determine.
- Structures of the array CSUs are emerging in recent years through state-of-the-art cryoEM/cryoET techniques, yet further developments are needed to achieve understanding of chemotaxis signaling at near-atomic level, with time-resolved structural snapshots during a real ligand-induced signaling transduction and signal adaption. A number of challenges remain, such as limitation of sample thickness in minicells and native intact cells, anisotropic resolution due to preferred orientation in both monolayer arrays and lysed bacterial ghost cells.
- New developments in cryoEM/cryoET, both in hardware such as focused ion beam milling [43], Falcon 4 direct detector with SelectrisX energy filter, cold field-emission gun [44,45] and potentially Cc corrector [46], and in cryoET STA software (Table 2), would potentially help to achieve high-resolution views of different signaling states, and even in a time-resolved manner. New sample systems and data collection/processing strategies, including CSU samples for cryoEM single particle methods, can be developed and implemented. The ultimate goal is to construct molecular movies of the chemotaxis signaling process using cryoEM/cryoET structural snapshots of individual signaling states.

## Open Access Statement

Open access for this article was enabled by the participation of University of Oxford in an all-inclusive Read & Publish pilot with Portland Press and the Biochemical Society under a transformative agreement with JISC.

## Abbreviations

CSU: core signaling unit
LBD: ligand binding domain
MCPs: methyl-accepting chemotaxis proteins
MD: molecular dynamics
MDFF: molecular dynamics flexible fitting
STA: subtomogram averaging
